# EHMTI-0324. Expression of high-mobility group box 1 in the cerebral cortex after cortical spreading depression

**DOI:** 10.1186/1129-2377-15-S1-F26

**Published:** 2014-09-18

**Authors:** T Takizawa, M Shibata, Y Kayama, T Shimizu, H Toriumi, T Ebine, A Koh, N Suzuki

**Affiliations:** 1 Department of Neurology, Keio University School of Medicine, Tokyo, Japan

## Introduction and aims

HMGB1 (high-mobility group box 1), which can serve as both a DNA-binding protein and a cytokine-like secretory molecule, has been implicated in the pathophysiological processes initiated by cortical spreading depression (CSD). Here, we examined the expression of HMGB1 at the RNA and protein levels in the cerebral cortex subjected to CSD.

## Methods

CSD was induced by applying 1 M KCl to the cerebral cortex in male C57BL/6 mice. The induction of CSD was monitored by recording DC potentials at an electrode close to the CSD induction site. To examine HMGB1 expression, immunohistochemistry and in situ hybridization were performed for 10 micrometers-thick sections prepared from the cerebral cortices exposed to CSD. Non-treated control mice and sham-operated mice were also investigated.

## Results

The basal expression of HMGB1 transcript and protein was identified in neurons and astrocytes with HMGB1 immunoreactivity being localized exclusively within the nucleus. At 3 hours after CSD induction, the HMGB1 transcription level was more marked in the cerebral cortex subjected to multiple CSD (5 times) compared to single CSD. At 24 hours after CSD induction, the HMGB1 transcriptional activity returned to the basal level. Meanwhile, HMGB1 immunoreactivity was recognized in the cytoplasm as well as within the nucleus in a small proportion of neurons in the cerebral cortex subjected to CSD.

## Conclusions

CSD causes the release of HMGB1 from the nucleus in cortical neurons. Transient transcriptional upregulation of HMGB1 is driven by CSD, apparently contributing to replenishment of the molecule after its release.

No conflict of interest.

